# UDP-Glucuronosyltransferase 1A Compromises Intracellular Accumulation and Anti-Cancer Effect of Tanshinone IIA in Human Colon Cancer Cells

**DOI:** 10.1371/journal.pone.0079172

**Published:** 2013-11-14

**Authors:** Miao Liu, Qiong Wang, Fang Liu, Xuefang Cheng, Xiaolan Wu, Hong Wang, Mengqiu Wu, Ying Ma, Guangji Wang, Haiping Hao

**Affiliations:** Key Lab of Drug Metabolism and Pharmacokinetics, State Key Lab of Natural Medicines, China Pharmaceutical University, Nanjing, China; Virginia Commonwealth University, United States of America

## Abstract

**Background and Purpose:**

NAD(P)H: quinone oxidoreductase 1 (NQO1) mediated quinone reduction and subsequent UDP-glucuronosyltransferases (UGTs) catalyzed glucuronidation is the dominant metabolic pathway of tanshinone IIA (TSA), a promising anti-cancer agent. UGTs are positively expressed in various tumor tissues and play an important role in the metabolic elimination of TSA. This study aims to explore the role of UGT1A in determining the intracellular accumulation and the resultant apoptotic effect of TSA.

**Experimental Approach:**

We examined TSA intracellular accumulation and glucuronidation in HT29 (UGT1A positive) and HCT116 (UGT1A negative) human colon cancer cell lines. We also examined TSA-mediated reactive oxygen species (ROS) production, cytotoxicity and apoptotic effect in HT29 and HCT116 cells to investigate whether UGT1A levels are directly associated with TSA anti-cancer effect. UGT1A siRNA or propofol, a UGT1A9 competitive inhibitor, was used to inhibit UGT1A expression or UGT1A9 activity.

**Key Results:**

Multiple UGT1A isoforms are positively expressed in HT29 but not in HCT116 cells. Cellular S9 fractions prepared from HT29 cells exhibit strong glucuronidation activity towards TSA, which can be inhibited by propofol or UGT1A siRNA interference. TSA intracellular accumulation in HT29 cells is much lower than that in HCT116 cells, which correlates with high expression levels of UGT1A in HT29 cells. Consistently, TSA induces less intracellular ROS, cytotoxicity, and apoptotic effect in HT29 cells than those in HCT116 cells. Pretreatment of HT29 cells with UGT1A siRNA or propofol can decrease TSA glucuronidation and simultaneously improve its intracellular accumulation, as well as enhance TSA anti-cancer effect.

**Conclusions and Implications:**

UGT1A can compromise TSA cytotoxicity via reducing its intracellular exposure and switching the NQO1-triggered redox cycle to metabolic elimination. Our study may shed a light in understanding the cellular pharmacokinetic and molecular mechanism by which UGTs determine the chemotherapy effects of drugs that are UGTs’ substrates.

## Introduction

UDP-glucuronosyltransferases (UGTs) catalyze the glucuronidation of many lipophilic endogenous substrates such as bilirubin and steroid hormones, and xenobiotics including carcinogens and clinical drugs [1], [2], [3]. In most cases, UGT-mediated metabolism promotes the metabolic elimination and diminishes the biological efficacies of the substrates, although several cases of bioactivation have been observed [4], [5]. UGTs are thus considered as an important detoxification system. Genetic polymorphisms of UGTs causing reduced enzyme activity have been associated with cancer risk, such as colorectal cancer, breast cancer, lung cancer, proximal digestive tract cancer, hepatocellular carcinoma, and prostate cancer [6], [7]. Alternatively, the enhanced enzymatic activities of UGTs may represent an important contributor to chemotherapeutic resistance of many drugs that are UGTs’ substrates, such as irinotecan, methotrexate, epirubicin, and tamoxifen [8], [9], [10], [11], implying a crucial role of UGTs in the anti-cancer therapy. UGTs are positively expressed in various types of tumor tissues and cells, albeit to a relatively lower level as compared with the corresponding normal tissues [12], [13], [14], [15]. Although UGTs have been claimed as an important cause of chemotherapeutic resistance, little is known about the direct influence of UGTs regarding the intracellular accumulation in the target cancer cells and chemotherapeutic efficacy of drugs.

Tanshinone IIA (TSA) is a diterpene phenanthrenequinone compound isolated from the dried root of salvia miltiorrhiza (Danshen in Chinese), which is a widely used herbal medicine with well proven cardiovascular and cerebrovascular efficacies [16], [17], [18]. In particular, accumulating evidence supports that TSA is a promising anti-cancer agent [19], [20], [21], [22]. Previously we have clarified that TSA is predominantly eliminated via sequential NAD(P)H: quinone oxidoreductase 1 (NQO1) and UGT catalyzed metabolism [23], [24]. NQO1 catalyzes a two-electron reduction of TSA producing a highly unstable catechol metabolite that can be swiftly glucuronidated if UGTs are present. However, when UGTs are absent, the highly reactive catechol intermediate can undergo a redox cycle of quinone reduction and auto-oxidation, a process that produces excessive amounts of reactive oxygen species (ROS). Based on this finding, we have recently validated that NQO1 is an important intracellular target of TSA that elicits the apoptotic death of human non-small cell lung cancer (NSCLC) cells [25].

On the basis of our recent finding that multiple UGT1A isoforms are involved in TSA glucuronidation [24], the present study focuses on elucidating the role of these UGTs in determining the intracellular accumulation and apoptotic effect of TSA in human colon cancer cells. Here we showed that TSA glucuronidation in UGT-positive cancer cells diminished TSA intracellular accumulation, broke NOQ1-triggered redox cycle, and consequently reduced TSA-induced ROS formation and its anti-cancer effect.

## Materials and Methods

### Cell Lines and Culture

Human colon cancer cell lines HT29 and HCT116 were obtained from the American Type Culture Collection (ATCC, USA). Cells grew in McCoy’s 5a (Gibco, USA) medium with 10% fetal bovine serum (Hyclone, USA), 100 U ml^−1^ penicillin, and 100 mg ml^−1^ streptomycin at 37°C in a humidified atmosphere with 5% CO_2_. For different purpose, cells were cultured for 24–72 hours in the medium and then drugs were added. Trypsin (2.5%) was used for cell harvest. All cells were mycoplasma free.

### Chemicals and Reagents

TSA was purchased from the National Institute for the Control of Pharmaceutical and Biological Products (Beijing, China), and prepared into solid dispersion with PEG6000 as described [26]. Propofol, 4-methylumbelliferone (4-MU), mycophenolic acid (MPA), N-acetyl cysteine (NAC), dicoumarol (DIC), glucose 6-phosphate, glucose 6-phosphate dehydrogenase, β-nicotinamide adenine dinucleotide phosphate (NADP), uridine 5′-diphosphate-glucuronic acid (UDPGA), D-saccharic acid 1,4-lactone, β-D-glucuronidase (Escherichia coli), chlorzoxazone, 2′, 7′-dichlorofluorescein diacetate (DCFH-DA), and 3-(4,5-dimethylthiazol-2-yl)-2,5-diphenyltetrazolium bromide (MTT) were all obtained from Sigma (St. Louis, MO, USA). Annexin V-FITC Apoptosis Detection Kit was purchased from Bipec Biopharma Corporation (USA). The real time PCR primers for detecting transcripts of UGT1A1, UGT1A3, UGT1A6, UGT1A9, and UGT1A10 were purchased from Invitrogen (CA, USA). Antibodies against UGT1A (Abcam, USA), UGT1A9 (Abcam, USA), and GAPDH (Boster Biology, China) were used in Western blot analysis. High performance liquid chromatography (HPLC) grade acetonitrile was obtained from Fisher Scientific (Toronto, Canada). All other chemicals were HPLC grade or the best grade that was commercially available.

### Transient Transfection of UGT1A siRNA

Transient transfection was performed using Lipofectamine RNAiMAX (invitrogen, USA) according to the manufacturer’s instruction. Briefly, the Stealth RNAi siRNA (invitrogen, USA) for UGT1A silence or a negative control was mixed with Lipofectamine RNAiMAX Reagent in Opti-MEM I (Gibco, USA) at a finial concentration of 20 nM, and the siRNA mixture was added to an appropriate culture plate at room temperature. Exponentially growing cells were subsequently seeded in the transfection mixture-containing plate. Cells were incubated in an incubator (5% CO_2_) at 37°C for 24–72 hours.

### Quantification of mRNA Levels

Total mRNA was extracted from cells, and cDNA was synthesized by the PrimeScrip RT reagent Kit (TaKaRa Biotechnology, Dalian, China). Quantitative real-time PCR was performed by SYBR Premix Ex Taq II (TaKaRa Biotechnology, Dalian, China) following the manufacturer’s instruction. The sequences of primers used in real-time PCR are listed on supporting information 1 (Table S1). PCR conditions were 95°C for 1 min, followed by 40 cycles of 95°C for 5 seconds, 60°C for 30 seconds, and 72°C for 30 seconds.

### Western Blot Analysis

Cells were harvested and total protein was extracted. The protein concentration was then determined using the BCA Protein Assay Kit (Beyotime, China). Equal amounts of protein (50 µg) were loaded and separated by SDS-PAGE electrophoresis. Proteins were then transferred to a PVDF membrane (PALL, USA). Blots were blocked with 5% skim milk in a TBST buffer and incubated for 24 hours at 4°C with specific primary antibodies. The membrane was washed 3 times with TBST and then incubated with HRP-conjugated secondary antibody (KeyGen, Nanjing, China) for 1 hour at 37°C. The signal was visualized by enhanced chemiluminescence (ECL, Millipore). The protein expression levels were normalized with GAPDH.

### UGT Activity Assay

UGT activity was presented by the substrate’s glucuronidation activity with cell S9 fractions. Cells were harvested by 2.5% trypsin and washed in ice-cold PBS, homogenized in PBS, and centrifuged at 9,000 g for 20 min at 4°C to obtain cell S9 fractions. The protein content of S9 fractions was determined using the BCA Protein Assay Kit (Beyotime, China). According to previous reports [27], [28], non-specific UGT1A substrate 4-MU was used to determine the general activity of UGT1A, and MPA, which is glucuronidated primarily by UGT1A9 was used to determine UGT1A9 activity. Briefly, 4-MU or MPA (0.5 mM) was incubated in a 200 µl reaction mixture containing 0.1 mg (0.2 mg for MPA) cell S9 fractions, 2 mM UDPGA, 1 mM saccharic acid 1,4-lactone, 5 mM MgCl_2_, and 50 mM Tris-HCl buffer (pH 7.4) at 37°C for 15 min (30 min for MPA). The S9 fractions were pretreated with alamethicin at a concentration of 25 µg mg^−1^ on ice for 20 min. After pre-incubation for 5 min at 37°C, the reaction was started by adding UDPGA. Reactions were stopped by an addition of 400 µl of ice-cold acetonitrile and samples were centrifuged for 10 min at 20,000 g. Supernatant samples (100 µl) were analyzed by a Shimadzu (Kyoto, Japan) LC-2010C HPLC system equipped with a quaternary pump, autosampler, column oven, and UV detector. The separation was performed using a Lunar-C18 column (250×4.6 mm i.d., 5 µm, Phenomenex Inc., China) with a guard column (Phenomenex Inc., China). For 4-Mu, the mobile phase was acetonitrile (A) and water with 25 mM K_2_HPO_3_ (B) at a flow rate of 1 ml min^−1^; elution was conducted with the following gradient: 15% A (0–2 min), linear gradient from 15% to 60% A (2–5 min), 60% to 40% A (5–8 min), and 15% A for 8–10 min, and then for another 5 min of equilibration with a column temperature of 40°C and UV detection at 322 nm. For MPA, the mobile phase was acetonitrile (A) and water with 0.1% (v/v) acetic acid (B) at a flow rate of 1 ml min^−1^; elution was performed with the following gradient: 45% (0–2 min), linear gradient from 45% to 80% A (2–8 min), 80% A for another 1 min, and 45% A for 9–10min and then for another 4 min of equilibration with a column temperature of 40°C and UV detection at 250 nm. Accurate quantitation of the newly formed glucuronides was achieved by calibrating with authentic standards (Sigma, USA).

### Glucuronidation Assay of TSA in S9 Fractions

The S9 fraction (0.25 mg) was incubated with indicated concentrations of TSA in a reaction mixture consisting of 2 mM UDPGA, 1 mM saccharic acid 1,4-lactone, 5 mM MgCl_2_, and an NADPH-regenerating system containing 0.2 mM NADP, 1.9 mM glucose 6-phosphate, 1.2 U ml^−1^ glucose-6-phosphate dehydrogenase, and 50 mM Tris-HCl buffer (pH 7.4) in a final volume of 200 µl. The S9 fraction was pretreated with alamethicin at a concentration of 25 µg mg^−1^ on ice for 20 min to diminish the latency of UGT activity. For enzyme kinetics assay, after pre-incubation for 5 min at 37°C, the reaction was started by adding UDPGA and incubated at 37°C for 60 min. For propofol inhibition study, propofol (0–400 µM) was co-incubated with TSA (20 µM) at 37°C for 20 min. All the reactions were terminated by ice-cold acetonitrile, followed by centrifugation at 20,000 g for 10 min to obtain the supernatants, and then analyzed by HPLC system the same as described above with the method based on our previous report [24].

### TSA Intracellular Accumulation and Glucuronidation in Living Cells

Exponentially growing cells with 70% confluence were exposed to 20 µM TSA for 0.5, 2, 6, 24 and 48 hours. Cells and culture medium were collected separately at indicated time points. Cells were washed for three times by ice-cold PBS. Ultrapure water (300 µl) was added to each cell sample and freezing/thawing for three times to break the cells. Either cell or culture medium sample (100 µl) was added with ice-cold acetonitrile (300 µl) and mixed by strong vortex for 5 min, followed by centrifugation at 20,000 g for 10 min to obtain the supernatant, then analyzed by the HPLC method based on our previous report [24]. Drug concentrations were normalized by determining the protein concentration of the cell samples using the BCA Protein Assay Kit (Beyotime, China).

### ROS Assay

Indicated concentration of TSA was administrated to cells at 70% confluence for 1 hour. Then cells were treated with DCFH-DA for 30 min and washed by ice-cold PBS for three times. Cellular ROS can converse non-fluorescent DCFH-DA to its fluorescent derivative DCF. ROS formation was analyzed by measuring the fluorescence intensity of DCF at 535 nm (with 488 nm excitation) in Synergy-H1 fluorimeter (Bio-Tek Instruments).

### Cytotoxicity Assay

Cells were seeded at 7,000 cells per well to a 96-well plate and incubated overnight. The cells were subsequently exposed to the indicated concentrations of TSA. After 48 hours (for HCT116) or 72 hours (for HT29), MTT (5 mg ml^−1^) was added to each well, and the plate was incubated at 37°C for another 4 hours. The MTT solution was then removed and 150 µL of DMSO was added per well. The absorbance at 570 nm was measured by a microplate reader.

### Apoptosis Assay

Cells were seeded by 2×10^5^/well into 6-well plate and reached 60% confluence after 72 hours culture. Then cells were exposed to the indicated concentration of TSA for 48 hours (HCT116) or 72 hours (HT29) and harvested by 0.25% trypsin without EDTA and washed by ice-cold PBS. Annexin V-FITC Apoptosis Detection Kit (Bipec Biopharma Corporation, USA) was used to stain the cells according to the manufacturer’s instruction. Samples were analyzed by using a flow cytometer (BD FACSCalibur, USA).

### Statistical Analysis

All data are presented as means ± SD of at least three independent experiments. Statistical differences between two groups were evaluated using the Student’s t-test; for multiple comparisons, one way analysis of variance followed by Dunnet test was applied. The difference was considered significant at *P<0.05, **P<0.01, or ***P<0.001.

## Results

### Multiple UGT1A Isoforms are Positively Expressed in HT29 but not in HCT116 Cells

**Figure 1:**
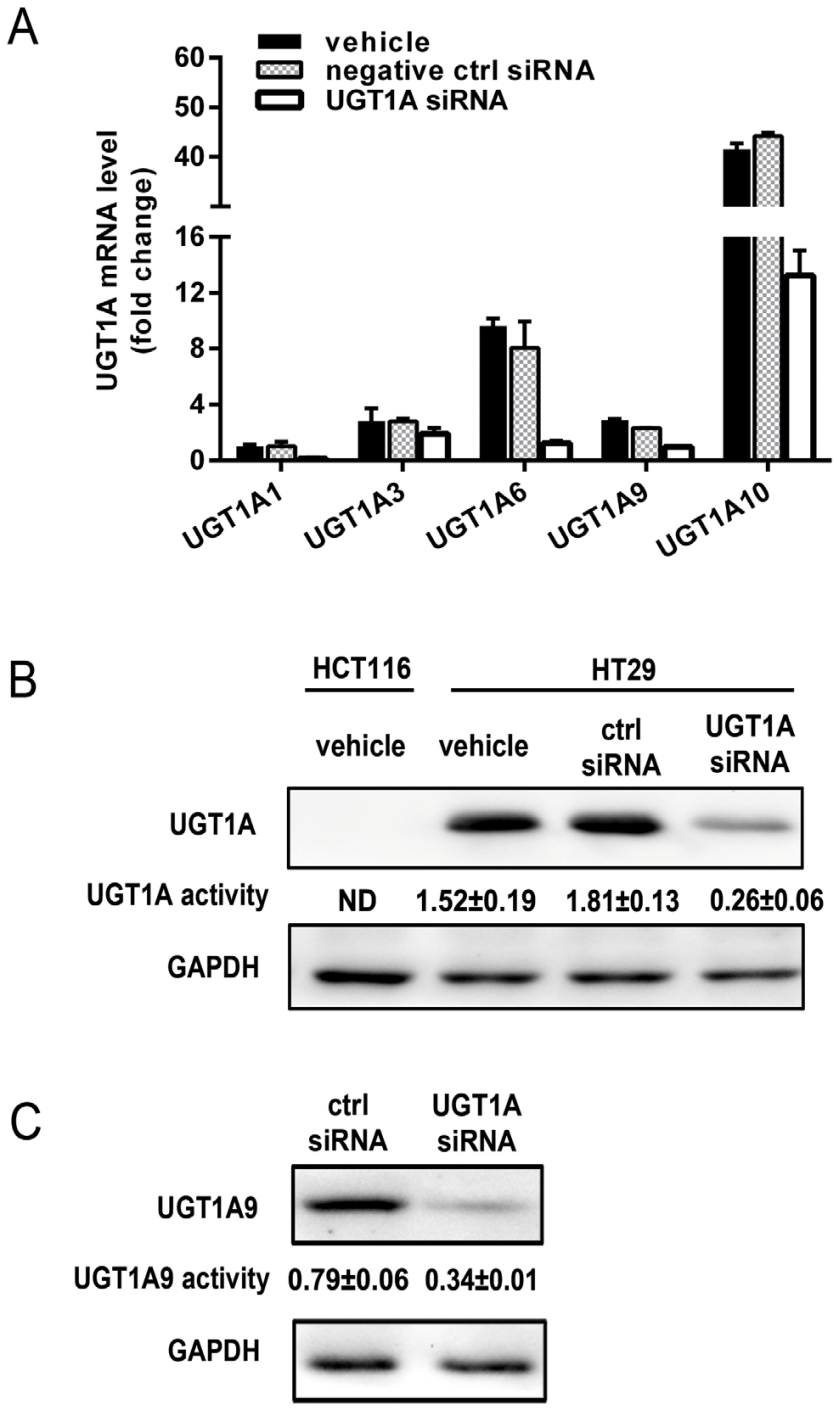
Multiple UGT1A isoforms are positively expressed in HT29 but not in HCT116 cells. Cells were pretreated with UGT1A siRNA, non-specific siRNA (negative control) or vehicle for 48 hours. (A) mRNA levels of UGT1A isoforms in HT29 cells. UGT1A1 mRNA level of the cells with vehicle was taken as 1; (B) protein levels and enzyme activities of UGT1A; (C) protein levels and enzyme activities of UGT1A9. The total UGT1A activity was determined by detecting the velocity of 4-Mu glucuronidation, and the UGT1A9 specific activity was determined by detecting the velocity of MPA glucuronidation. The enzyme activity was expressed as nmol per min per mg protein. A UGT1A activity <0.1 nmol min^−1 ^mg^−1^ was considered nondetectable (ND). Results are presented as mean ± SD of at least three independent experiments.

We first evaluated the expression levels of UGT1A isoforms which were involved in TSA glucuronidation by real time PCR in both HT29 and HCT116 cell lines. Figure 1A shows the gene expression pattern of UGT1A isoforms in HT29 cells. In contrast, no expression of UGT1A genes was detected in HCT116 cells (data not shown). To further investigate the role of UGTs, specific siRNA was used to silence UGT1A genes in HT29 cells. Three pairs of siRNAs directed against the UGT1A sequence were designed, and the best pair with the highest silencing effects along with non-specific siRNA as a negative control was examined. After siRNA transfections, mRNA levels were evaluated in HT29 cells. The mRNA levels of UGT1A1, UGT1A3, UGT1A6, UGT1A9, and UGT1A10 were reduced by 85.8%, 31.4%, 87.5%, 66.5%, and 68.2%, respectively, while the negative control siRNA had little effect (Figure 1A). Western blot assay supported a high expression level of UGT1A in HT29 cells, whereas no detectable UGT1A protein was observed in HCT116 cells (Figure 1B). The protein expression of total UGT1A and specific UGT1A9 was sharply decreased by UGT1A siRNA in HT29 cells (Figure 1B and 1C). The results of UGT activity assay showed that HT29 cells possess high capacity towards the glucuronidation of 4-MU, a general UGT1A substrate, and MPA, a relatively specific UGT1A9 probe. 4-MU and MPA glucuronidation activities were decreased with UGT1A siRNA transfection by 85.6% and 57%, respectively (Figure 1B and 1C). Consistent with mRNA and protein levels examination, no UGT1A specific enzymatic activity was detected in HCT116 cells (Figure 1B).

### The Inhibition of UGT1A Expression or UGT1A9 Activity Reduces TSA Glucuronidation in HT29 Cell S9 Fractions

**Figure 2:**
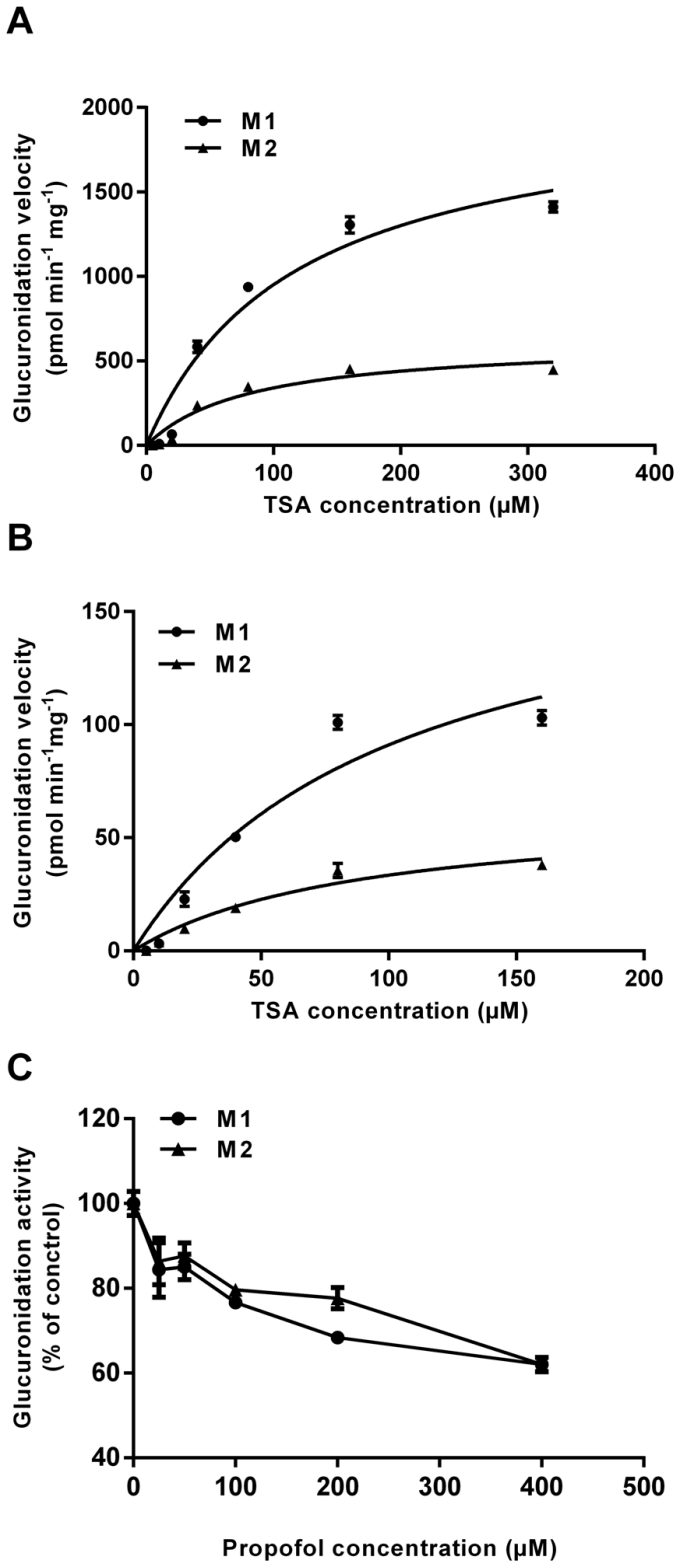
TSA glucuronidation in HT29 cell S9 fractions. Enzyme kinetics for the formation of TSA glucuronides (M1 and M2) was examined in (A) cell S9 fractions prepared from HT29 cells pretreated with negative control siRNA, and (B) cell S9 fractions prepared from HT29 cells pretreated with UGT1A siRNA. (C) inhibitory effect of propofol (0–400 µM) on TSA glucuronidation in HT29 cell S9 fractions. Results are presented as mean ± SD of three independent experiments.

To gain understanding of TSA glucuronidation by colon cancer cells, we performed the enzyme kinetic assay using S9 fractions prepared from HT29 cells with or without UGT1A siRNA treatment. Consistent with our previous study [24], M1 and M2, a pair of regioisomers of TSA catechol glucuronides, were detected from HT29 but not HCT116 cell S9 fractions. TSA glucuronidation displayed a typical Michaelis-Menten kinetics (Figure 2A and 2B). Kinetic parameters, including the apparent K_m_, maximum velocity (V_max_), intrinsic clearance (CL_int_, V_max_/K_m_) for M1 and M2, and sum CL_int_ (M1+M2) are summarized in Table 1. The silencing of UGT1A isoforms by UGT1A siRNA leaded to a about 10-fold decrease of V_max_ values for the production of both M1 and M2, while had little influence in K_m_ values. Accordingly, the CL_int_ for M1 and M2 and the sum CL_int_ (M1+M2) of the UGT1A silence group were approximately 10-fold lower than those of the negative control group.

**Table 1:** Best-fit enzyme kinetics parameters for the formation of TSA glucuronides (M1 and M2) in HT29 cell S9 fractions.

	K_m_ (µM)		V_max_ (pmol min^−1 ^mg^−1^)		CL_int_ (µl min^−1 ^mg^−1^)		CL_int_ (M1+M2) (µl min^−1^ mg^−1^)
	M1	M2	M1	M2	M1	M2	
Negative control	116.7±4.4	82.4±4.2	2059.3±40.5	620.6±20.4	17.6±0.31	7.5±0.15	25.2±0.34
UGT1A-silence	102.1±9.9	89.7±3.3	184.1±12.7***	63.4±1.2***	1.8±0.05***	0.7±0.03***	2.5±0.07***

Data are shown as mean ± SD of three independent experiments;

*** p<0.001, UGT1A-silence group vs negative control group.

The inhibitory effect of propofol on TSA glucuronidation in HT29 cell fractions was also examined. As a UGT1A9 specific substrate [8], propofol showed approximate 25% inhibition of both M1 and M2 at 100 µM, and around 40% inhibition at 400 µM (Figure 2C).

### UGT1A Determinates TSA Accumulation in Colon Cancer Cells

**Figure 3:**
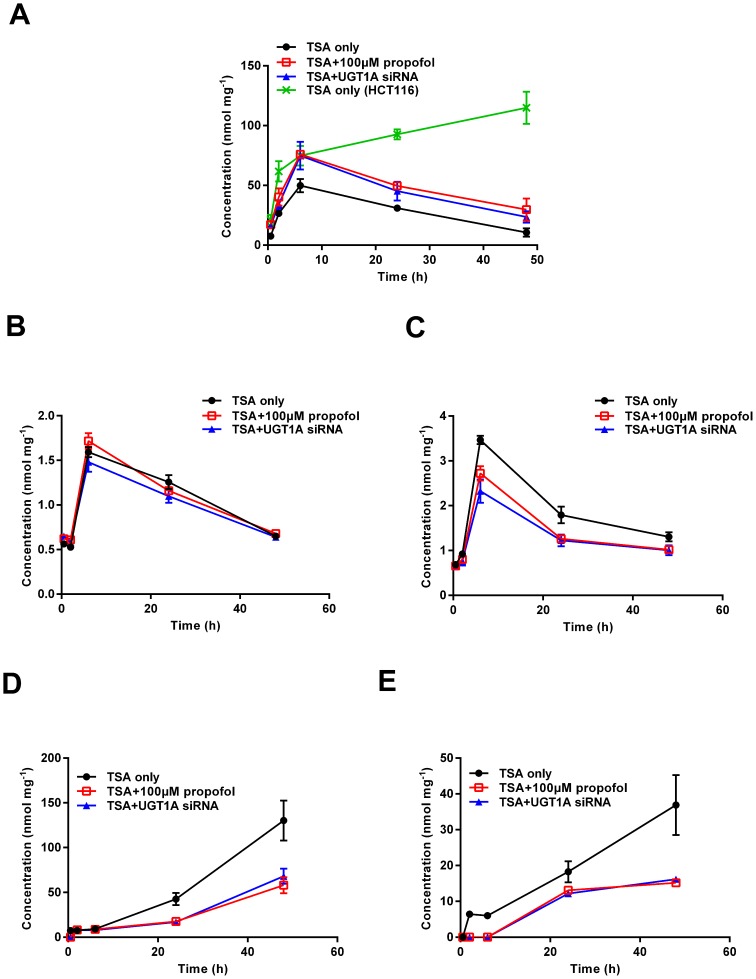
TSA intracellular accumulation and glucuronidation profile in colon cancer cells. HT29 cells were pretreated with UGT1A siRNA or vehicle for 48 hours, or pretreated with propofol (100 µM) for 1 hour. Then, cells were exposed to TSA (20 µM) for 0.5, 2, 6, 24, and 48 hours and samples of both the culture medium and cells were collected and prepared for HPLC analysis. TSA and its glucuronides (M1 and M2) were detected with the method based on our previous report [24]. (A) intracellular TSA of HT29 or HCT116 cells; (B) intracellular M1 of HT29 cells; (C) intracellular M2 of HT29 cells; (D) M1 in HT29 cell culture medium; (E) M2 in HT29 cell culture medium. Data are shown as mean ± SD of at least three independent experiments.

To test whether UGT1A can influence TSA disposition in the living cells, we performed a cellular pharmacokinetic study. The dynamic intracellular accumulation of TSA and its metabolites (M1 and M2) were determined. Of interest, the intracellular level of TSA continuously increased over the course of 48 hours following TSA treatment in HCT116 cells. However, TSA concentration in HT29 cells peaked at 6 hours and then dramatically decreased (Figure 3A). The area under curve from 0 to 48 hours (AUC_0–48 h_) and maximum concentration (C_max_) of TSA in HCT116 were much higher than that in HT29 cells (Table 2). Pretreatment of HT29 cells with propofol resulted in a significant increasing intracellular accumulation of TSA. Similarly, UGT1A siRNA transfection also increased the TSA accumulation in HT29 cells (Figure 3A, Table 2). Both M1 and M2 were detectable in HT29 cells at 0.5 hour after TSA treatment, suggesting a rapid intracellular production of glucuronides. The intracellular levels of M1 and M2 peaked at 6 h and then decreased (Figure 3B and 3C), while the levels of TSA glucuronides in culture medium accumulated continuously over the course of detection (Figure 3D and 3E). The formation of M2, but not M1, was reduced by either propofol or UGT1A siRNA transfection in HT29 cells (Figure 3B and 3C, Table 2). Both propofol and UGT1A siRNA significantly decreased the formation of TSA glucuronides M1 and M2 in the culture medium (Figure 3D and 3E, Table 2).

**Table 2:** AUC_0–48 h_ and C_max_ values of TSA and its glucuronides (M1 and M2) in colon cancer cells and in the cell culture medium.

		AUC_0–48 h_ (h nmol mg^−1^)			C_max_ (nmol mg^−1^)		
		TSA only	TSA+100 µMpropofol	TSA+UGT1AsiRNA	TSA only	TSA+100 µMpropofol	TSA+UGT1AsiRNA
In cells	TSA(HT29)	1404.1±104.9	2356.9±153.8**	2160.6±328.2**	49.8±5.5	75.9±1.2*	74.8±11.6*
	TSA(HCT116)	4333.8±257.4***	–	–	114.9±13.4***	–	–
	M1(HT29)	52.7±3.2	53.1±2.3	48.4±3.9	1.6±0.06	1.7±0.09	1.5±0.11
	M2(HT29)	92.5±4.6	71.0±4.9**	64.5±8.5***	3.5±0.09	2.7±0.16**	2.3±0.26***
In medium	M1(HT29)	2819.1±0.7	1237.5±78.5***	1300.5±103.9**	130.2±22.3	58.0±8.9**	68.2±8.4**
	M2(HT29)	999.1±36.6	458.6±8.4**	451.9±5.9**	36.9±8.4	15.2±0.5**	16.2±0.5**

Data are shown as mean ± SD of three independent experiments;

* p<0.05 **p<0.01 ***p<0.001, propofol pretreatment (HT29) or UGT1A siRNA pretreatment (HT29) or TSA only (HCT116) group vs TSA only (HT29) group.

### UGT1A Diminishes TSA-induced ROS Formation

**Figure 4:**
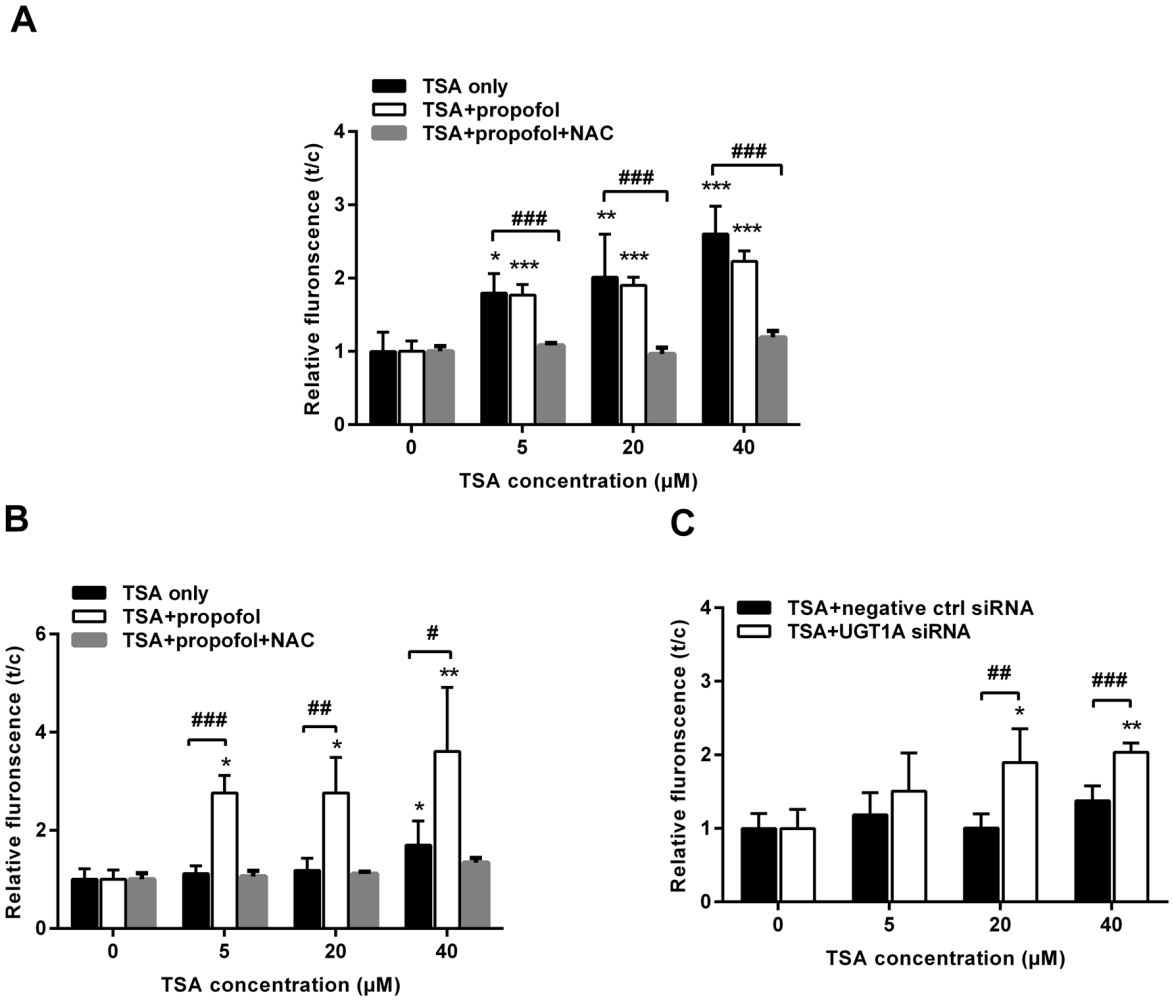
TSA Induces ROS in colon cancer cells. Cells were pretreated with UGT1A siRNA or non-specific siRNA (negative control) for 48 hours, or pretreated with propofol (100 µM)/NAC (5 mM) for 1 hour. Then, cells were exposed to TSA (5, 20, 40 µM) for 1 hour and subsequently treated with DCFH-DA. The fluorescence intensity was measured by a fluorimeter. (A) HCT116 cells; (B) and (C) HT29 cells. Results are presented as mean ± SD of at least four independent experiments (*P<0.05, **P<0.01, ***P<0.001, TSA treatment vs control cells; ^#^P<0.05, ^##^P<0.01, ^###^P<0.001, propofol/NAC pretreatment vs TSA only, or UGT1A siRNA pretreatment vs negative control siRNA pretreatment).

We have previously shown that TSA undergoes NQO1 metabolism to produce a highly unstable catechol intermediate which could be either conjugated by UGTs or spontaneously reverted back to parent TSA to form a futile redox cycle with excessive amounts of ROS production [23]. Moreover, we found that TSA produced a significant level of ROS in NSCLC cells, a NQO1 positive and UGT negative cell line [25]. We thus reasoned that, in the presence of UGT1A, the TSA-triggered redox cycle can be switched to metabolic elimination and thereby reducing the production of ROS. To examine this hypothesis, the DCF staining assay was conducted to monitor TSA-induced ROS formation. TSA induced dose-dependent formation of ROS in HCT116 cells (Figures 4A). However, these changes in ROS formation were not observed in HT29 cells (Figure 4B). When propofol was used to inhibit UGT1A9 activity in HT29 cells, the TSA-induced ROS level was significantly increased by approximately 3.6-fold at 40 µM TSA, and this increase was reversed by NAC (Figure 4B). In contrast, propofol did not change the TSA-induced ROS level in HCT116 cells, but the combination of NAC still declined TSA-induced ROS level (Figures 4A). In addition, UGT1A siRNA transfection also significantly enhanced ROS levels by 2.0–fold at 40 µM in HT29 cells (Figure 4C).

### UGT1A Causes the Resistance of Colon Cancer Cells to TSA-induced Cytotoxicity

**Figure 5:**
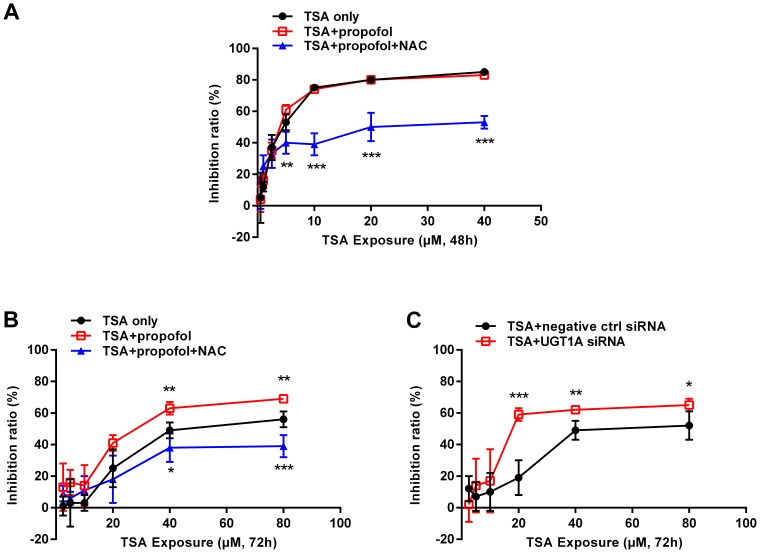
UGT1A causes the resistance of colon cancer cells to TSA-induced cytotoxicity. Cells were pretreated with UGT1A siRNA or non-specific siRNA (negative control) for 24 hours, or pretreated with propofol (100 µM)/NAC (5 mM) for 1 hour. Then, Cells were exposed to gradient concentrations of TSA (2.5–80 µM for HT29; 0.5–40 µM for HCT116) for indicated time and MTT assay was performed. (A) HCT116 cells; (B) and (C) HT29 cells. Results are presented as mean ± SD of at least four independent experiments (*P<0.05, **P<0.01, ***P<0.001).

Excess ROS can induce disruption of intracellular redox homeostasis, and irreversible oxidative modifications of lipid, protein, or DNA, subsequently promote cell apoptotic death via both death receptor and mitochondria-mediated pathways [29]. We have recently validated that TSA-induced cytotoxicity is ROS dependent [25]. Since the presence of UGT1A in HT29 cells compromised the production of ROS, we propose that the expression of UGT1A genes would increase the resistance of cancer cells to TSA-induced cytotoxicity. To this end, MTT assay was performed in both HT29 and HCT116 cells. The results showed that TSA produced dramatic cytotoxicity in HCT116 cells expressing no UGT1A with an IC_50_ value at 4.5±0.4 µM (Figure 5A). In contrast, HT29 cells, expressing abundant UGT1A enzymes, were found highly resistant to TSA cytotoxicity with an IC_50_ value at 54.3±4.7 µM (Figure 5B). Pretreatment with propofol to inhibit UGT1A9 activity significantly increased TSA cytotoxicity in HT29 cells with an IC_50_ value at 29.9±4.5 µM (Figure 5B), which was reversed by the combination of NAC (IC_50_>80 µM). In HCT116 cells, propofol-enhanced TSA cytotoxicity was not observed, but NAC combination caused high TSA resistance (Figure 5A). Together, these results suggest that the effect of propofol in HT29 cells is from the inhibition of UGT1A9 and thereby promoting the futile redox cycle of TSA. Consistently, UGT1A siRNA transfections also enhanced the cytotoxic effect of TSA in HT29 cells and significantly reduced the IC_50_ value to 25.9±5.6 µM (Figure 5C).

### UGT1A Compromises TSA-induced Apoptosis of Colon Cancer Cells

**Figure 6:**
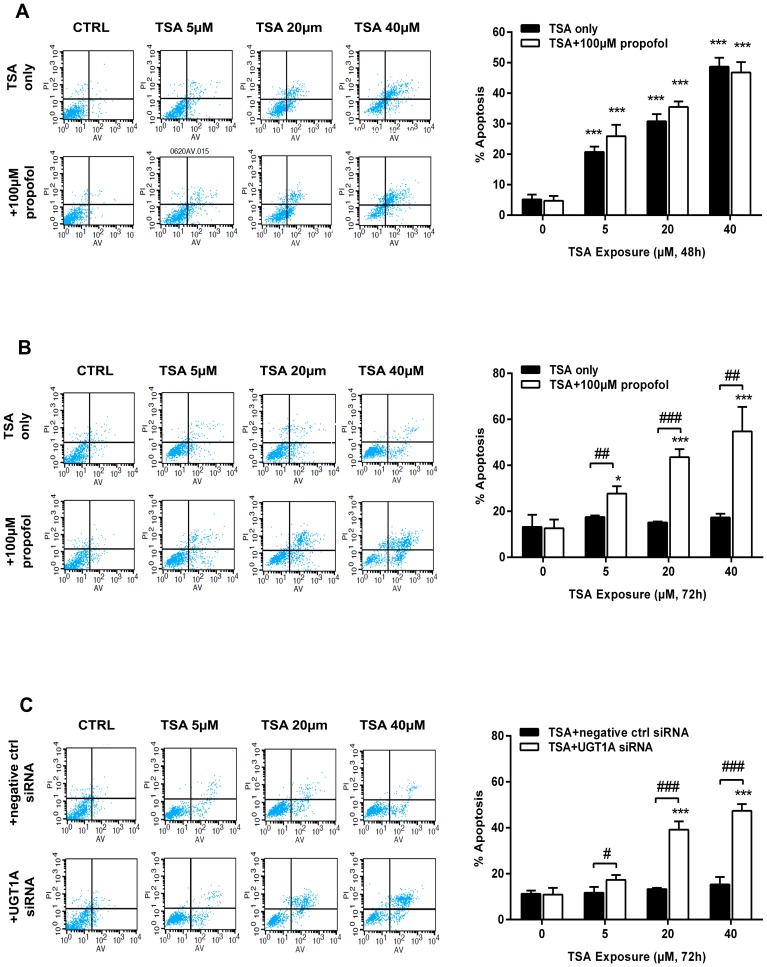
UGT1A compromises TSA-induced apoptosis of colon cancer cells. Cells were pretreated with UGT1A siRNA or non-specific siRNA (negative control) for 72 hours, or pretreated with propofol (100 µM) for 1 hour. Then, cells were exposed to TSA (5, 20, 40 µM) for indicated time and collected. Cells were stained by Annexin V-FITC/PI and examined by a flow cytometry. (A) HCT116 cells; (B) and (C) HT29 cells. Results are presented as mean ± SD of at least three independent experiments (*P<0.05, **P<0.01, ***P<0.001, TSA treatment vs control cells; ^#^P<0.05, ^##^P<0.01, ^###^P<0.001, propofol pretreatment vs TSA only, or UGT1A siRNA pretreatment vs negative control siRNA pretreatment).

To further explore the role of total UGT1A and UGT1A9 in TSA-mediated anti-cancer activity, cell apoptotic death was examined by the Annexin V-FITC/PI staining assay. When HT29 cells were exposed to the indicated concentration of TSA for 72 hours, there were few detectable apoptotic cells (Figure 6B). However, apoptotic death was observed in HCT116 cells after 48 hours of TSA exposure in a dose-dependent manner. Apoptotic cell death was 20.7±1.7%, 30.8±2.4%, and 48.7±3.0% with TSA concentrations of 5, 20, and 40 µM, respectively (Figure 6A). Propofol (100 µM) significantly restored the susceptibility of HT29 cells to TSA-induced apoptotic death, from the basal apoptotic levels to an apoptotic ratio of 27.8±4.5%, 45.0±3.5%, and 53.5±14.7% at 5, 20, and 40 µM TSA, respectively (Figure 6B). Propofol-enhanced apoptosis was not observed in HCT116 cells (Figure 6A). Similarly, UGT1A siRNA transfection increased TSA-induced cell apoptosis in HT29 cells, characterizing with 16.2±1.4%, 40.3±4.3%, and 48.5±3.2% apoptotic cells at TSA concentrations of 5, 20, and 40 µM, respectively (Figure 6C).

## Discussion

Many anti-cancer agents are substrates of UGTs, and for this reason, the functional significance of UGTs in causing chemotherapeutic resistance has become an important concern. In spite of previous efforts in addressing this important issue, little is known regarding the direct effect of UGTs expressed in tumor tissues/cells in determining the intracellular accumulation and the resultant anticancer effect of such agents that are UGTs’ substrates. We show in the present study that the expression level of UGT1A, and in particular UGT1A9, is an important factor in determining the intracellular accumulation and the apoptotic effect of TSA in human colon cancer cells.

Our previous study identified that UGT1A isoforms, including UGT1A1, UGT1A3, UGT1A6, UGT1A10, and in particular UGT1A9, are the dominant enzymes involved in the glucuronidation of TSA following its reduction by NQO1. Although UGT2B7 may be involved in the production of M1 and contribute to the total glucuronidation of TSA, the CL_int_ (M1+M2) value of UGT1A9 was much higher than that of UGT2B7 [24]. And in HT29 cells, the basal expression of UGT2B7 is much lower than UGT1A9 (Figure S1). Based on this finding, the silencing of UGT1A by UGT1A siRNA and the inhibiting of UGT1A9 by propofol were examined in TSA metabolism and toxicity in the present study.

HT29 cells possess sufficient UGT enzyme activity to glucuronidate TSA in both the S9 fractions and living cells (Figure 1, 2, 3). The enzyme kinetic assay showed that cells treated with UGT1A siRNA did not change the K_m_ values of M1 and M2, but V_max_ and CL_int_ values were significantly decreased (Figure 2, Table 1), indicating that the silencing of UGT1A isoforms is unlikely to affect the affinity of the UGT1A enzymes towards TSA, but can dramatically decrease the UGT1A enzyme activity due to the reduced levels of UGT1A proteins. In living cells, the intracellular accumulation of TSA was obviously different between HT29 and HCT116 cells. Compared with HCT116 cells, HT29 cells displayed a significant lower AUC value of TSA, suggesting that UGT1A is an important determinant of the exposure concentration and time of TSA in target cells. The pretreatment of HT29 cells with either UGT1A siRNA or propofol significantly enhanced the intracellular accumulation of TSA, characterized with much higher C_max_ and AUC values. Moreover, the levels of both M1 and M2 were much lower in the culture medium of cells pretreated with either UGT1A siRNA or propofol. Of interest, the concentration of M1 and M2 in the medium kept increasing during the experimental process, hinting to a role of UGTs in promoting the metabolic elimination of TSA from the target cells to the circulating system (Figure 3, Table 2).

Our recent finding has indicated that NQO1 is the main intracellular anti-cancer target of TSA in UGT deficient NSCLC cells [25]. In line with this finding, MTT assays indicated that either NQO1 siRNA or NQO1 inhibitor dicoumarol (DIC, 5 µM) could also reduce the TSA cytotoxicity in colon cancer HT29 and HCT116 cells (Figure S2). NQO1 triggers a TSA-induced redox cycle that is considered the dominant mechanism by which TSA induces apoptotic NSCLC cell death. Because glucuronidation may break such a redox cycle by diverting to the production of stable TSA glucuronides, we thus examined the potential influence of UGT1A in TSA-induced ROS production in human colon cancer cells. In accord with our previous finding in NSCLC cells, TSA induced a concentration dependent production of ROS in HCT116 cells, while DIC pretreatment could dramatically reduce the ROS production in HCT116 cells (Figure S3). However, no significant production of ROS was observed in HT29 cells (Figure 4B). Of interest, the pretreatment of HT29 cells with either UGT1A siRNA or propofol significantly recovered the ability of colon cancer cells in producing ROS upon TSA treatment, and this recovery was compromised by NAC, which is a ROS scavenger (Figure 4B and 4C). These results strongly indicate that UGT1A protein expression in HT29 cells efficiently break the NQO1-triggered redox cycle and divert TSA metabolism from the redox cycle to the metabolic elimination. Moreover, the presence of glucuronidation strongly decreased the intracellular accumulation of TSA in HT29 cells (Figure 3, Table 2). Thus, it is reasonable to predict that HT29 cells may be less sensitive to TSA-induced cytotoxicity than HCT116 cells. As expected, the sensitivity of HCT116 cells to TSA-induced cytotoxicity was approximately10-fold higher than that of HT29 cells (Figure 5A and 5B), although the expression and activity of NQO1 were higher in HT29 than those in HCT116 cells (Figure S4). Either UGT1A siRNA or propofol pretreatment could significantly sensitize HT29 cells to TSA-induced cytotoxicity (Figure 5B and 5C). NAC could reverse propofol-enhanced TSA cytotoxicity, verifying the important role of ROS in the cell death process triggered by TSA (Figure 5B and 5C). The apoptotic assay further supports a role for glucuronidation in determining the sensitivity of human colon tumor cells to the anti-cancer efficacy of TSA (Figure 6). Of note, the percentage of apoptotic cell death in the flow cytometry apoptosis analysis was much lower than that of the total cell death observed in MTT assay. This discrepancy suggests that the possibility that other types of cell death, such as necrosis, may be responsible for TSA induced cytotoxicity, although different cell density in the two experiments may be an important cause for this discrepancy.

**Figure 7:**
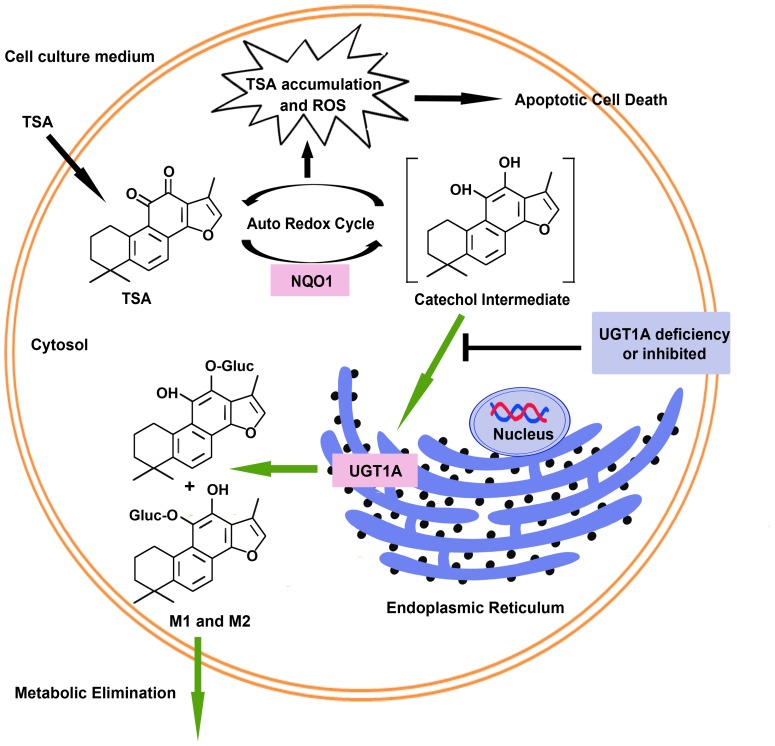
UGT1A determines intracellular accumulation and the resultant apoptotic cell death of TSA in human colon cancer cells.

In conclusion, we have validated that the expression and activity of UGT1A are important determinants towards the intracellular accumulation and the resultant apoptotic effect of TSA in colon cancer cells. Lack of glucuronidation renders a continuous redox cycle of TSA reduction and auto-oxidization that produces dramatic intracellular production of ROS and finally leads to apoptotic cell death. In contrast, the presence of high levels of UGT1A activity, in particular UGT1A9 activity, may break this cycle and promote the metabolic elimination of TSA in cancer cells. The present study together with our previous findings suggests that the cytotoxicity of TSA depends on the balance of expression and activity of NQO1 and UGT1A (Figure 7). In addition, it is interesting to note that in tumor tissues the expression of NQO1 is much higher [30], [31], [32], [33] while UGTs are lower [12], [13], [14] than that in surrounding normal tissues. This property confers a high tumor-selectivity and low normal tissue toxicity on TSA and other similar agents such as β-lapachone [3], [34], which target NQO1 against tumor tissues while sparing normal tissues.

## Supporting Information

Figure S1 **UGT1A9 and UGT2B7 mRNA levels in HT29 cells.** mRNA levels were determined by RT-real time PCR. UGT2B7 mRNA level of HT29 cells were taken as 1.
(TIF)

Figure S2 **NQO1 affects TSA-induced cytotoxicity in HT29 and HCT116 cells.** Cells were seeded by 5000/well in 96-well plate with NQO1 siRNA or non-specific siRNA (negative control) and cultured for 24 hours. Or cells were seeded by the same density, cultured for 24 hours, and pretreated with dicoumarol (DIC, Sigma, USA) for 2 hour. Then, Gradient concentrations of TSA (2.5–80 µM for HT29; 0.5–40 µM for HCT116) were added to cell culture medium and incubated for the indicated time and subsequently MTT assay was performed. (A) and (B) HCT116 cells; (C) and (D) HT29 cells. Results are presented as mean ± SD of at least four independent experiments (*P<0.05, **P<0.01, ***P<0.001).
(TIF)

Figure S3 **DIC inhibits TSA-induced ROS formation in HCT116 cells.** Cells were pretreated with DIC (5 µM) for 2 hours. Then, cells were exposed to TSA (5, 20, 40 µM) for 1 h and subsequently treated by DCFH-DA. The fluorescence intensity was detected by a fluorimeter. Results are presented as mean ± SD of at least three independent experiments (*P<0.05, **P<0.01, ***P<0.001, TSA treatment vs control cells; ^#^P<0.05, ^##^P<0.01, ^###^P<0.001, DIC pretreatment vs TSA only).
(TIF)

Figure S4 **NQO1 protein levels and enzyme activities were determined in HT29 and HCT116 cells.** NQO1 siRNA was used for NQO1-silence in both HT29 and HCT116 cells. Non-special siRNA was added as negative control. Specific NQO1 enzyme activity was determined as the rate of DIC-inhibitable 2, 6-Dichlorophenolindophenol (DCPIP, Sigma, USA) reduction in cell S9 fractions. The reaction was started by the addition of DCPIP, and the reduction of DCPIP was measured at room temperature at 600 nm by a microplate reader. The DIC-inhibitable part of DCPIP reduction was used to calculate NQO1 activity expressed as nmol DCPIP per minute per mg protein. Results are presented as mean ± SD of at least three independent experiments.
(TIF)

Table S1 **Sequences of the primers used in the study.**
(DOCX)

## References

[1] Tukey RH, Strassburg CP (2000) Human UDP-glucuronosyltransferases: metabolism, expression, and disease. Annu Rev Pharmacol Toxicol 40: 581–616. 10836148 10.1146/annurev.pharmtox.40.1.581

[2] Bushey RT, Dluzen DF, Lazarus P (2013) Importance of UDP-glucuronosyltransferases 2A2 and 2A3 in tobacco carcinogen metabolism. Drug Metab Dispos 41: 170–179. 23086198 10.1124/dmd.112.049171 PMC3533432

[3] Cheng X, Liu F, Yan T, Zhou X, Wu L, et al (2012) Metabolic Profile, Enzyme Kinetics, and Reaction Phenotyping of beta-Lapachone Metabolism in Human Liver and Intestine in Vitro. Mol Pharm 9: 3476–3486. 23134532 10.1021/mp300296m

[4] Tang Y, LeMaster DM, Nauwelaers G, Gu D, Langouet S, et al (2012) UDP-glucuronosyltransferase-mediated metabolic activation of the tobacco carcinogen 2-amino-9H-pyrido[2,3-b]indole. J Biol Chem 287: 14960–14972. 22393056 10.1074/jbc.M111.320093 PMC3340249

[5] Southwood HT, DeGraaf YC, Mackenzie PI, Miners JO, Burcham PC, et al (2007) Carboxylic acid drug-induced DNA nicking in HEK293 cells expressing human UDP-glucuronosyltransferases: role of acyl glucuronide metabolites and glycation pathways. Chem Res Toxicol 20: 1520–1527. 17880178 10.1021/tx700188x

[6] Desai AA, Innocenti F, Ratain MJ (2003) UGT pharmacogenomics: implications for cancer risk and cancer therapeutics. Pharmacogenetics 13: 517–523. 12893990 10.1097/01.fpc.0000054116.14659.e5

[7] Nagar S, Remmel RP (2006) Uridine diphosphoglucuronosyltransferase pharmacogenetics and cancer. Oncogene 25: 1659–1672. 16550166 10.1038/sj.onc.1209375

[8] Cummings J (2003) Glucuronidation as a Mechanism of Intrinsic Drug Resistance in Human Colon Cancer: Reversal of Resistance by Food Additives. Cancer Res 63: 8443–8450. 14679008

[9] Almagro MCd (2011) UDP-glucuronosyltransferase 1A6 overexpression in breast cancer cells resistant to methotrexate. Biochemical Pharmacology 81: 60–70. 20854796 10.1016/j.bcp.2010.09.008

[10] Innocenti F, Iyer L, Ramirez J, Green MD, Ratain MJ (2001) Epirubicin glucuronidation is catalyzed by human UDP-glucuronosyltransferase 2B7. Drug Metab Dispos 29: 686–692. 11302935

[11] Sun D, Sharma AK, Dellinger RW, Blevins-Primeau AS, Balliet RM, et al (2007) Glucuronidation of active tamoxifen metabolites by the human UDP glucuronosyltransferases. Drug Metab Dispos 35: 2006–2014. 17664247 10.1124/dmd.107.017145

[12] Starlard-Davenport A, Lyn-Cook B, Radominska-Pandya A (2008) Identification of UDP-glucuronosyltransferase 1A10 in non-malignant and malignant human breast tissues. Steroids 73: 611–620. 18374377 10.1016/j.steroids.2008.01.019 PMC2408449

[13] Strassburg CP, Manns MP, Tukey RH (1997) Differential down-regulation of the UDP-glucuronosyltransferase 1A locus is an early event in human liver and biliary cancer. Cancer Res 57: 2979–2985. 9230212

[14] Izumi K, Li Y, Ishiguro H, Zheng Y, Yao JL, et al.. (2012) Expression of UDP-glucuronosyltransferase 1A in bladder cancer: Association with prognosis and regulation by estrogen. Mol Carcinog. 10.1002/mc.21978 23143693

[15] Nakamura A, Nakajima M, Yamanaka H, Fujiwara R, Yokoi T (2008) Expression of UGT1A and UGT2B mRNA in human normal tissues and various cell lines. Drug Metab Dispos 36: 1461–1464. 18480185 10.1124/dmd.108.021428

[16] Fu J, Huang H, Liu J, Pi R, Chen J, et al (2007) Tanshinone IIA protects cardiac myocytes against oxidative stress-triggered damage and apoptosis. Eur J Pharmacol 568: 213–221. 17537428 10.1016/j.ejphar.2007.04.031

[17] Han JY, Fan JY, Horie Y, Miura S, Cui DH, et al (2008) Ameliorating effects of compounds derived from Salvia miltiorrhiza root extract on microcirculatory disturbance and target organ injury by ischemia and reperfusion. Pharmacol Ther 117: 280–295. 18048101 10.1016/j.pharmthera.2007.09.008

[18] Jiang B, Zhang L, Wang Y, Li M, Wu W, et al (2009) Tanshinone IIA sodium sulfonate protects against cardiotoxicity induced by doxorubicin in vitro and in vivo. Food Chem Toxicol 47: 1538–1544. 19358873 10.1016/j.fct.2009.03.038

[19] Lu Q, Zhang P, Zhang X, Chen J (2009) Experimental study of the anti-cancer mechanism of tanshinone IIA against human breast cancer. Int J Mol Med 24: 773–780. 19885617 10.3892/ijmm_00000291

[20] Wang J, Wang X, Jiang S, Yuan S, Lin P, et al (2007) Growth inhibition and induction of apoptosis and differentiation of tanshinone IIA in human glioma cells. J Neurooncol 82: 11–21. 16955220 10.1007/s11060-006-9242-x

[21] Zhang Z, Gao J, Wang Y, Song T, Zhang J, et al (2009) Tanshinone IIA triggers p53 responses and apoptosis by RNA polymerase II upon DNA minor groove binding. Biochem Pharmacol 78: 1316–1322. 19591811 10.1016/j.bcp.2009.06.110

[22] Su CC, Lin YH (2008) Tanshinone IIA down-regulates the protein expression of ErbB-2 and up-regulates TNF-alpha in colon cancer cells in vitro and in vivo. Int J Mol Med 22: 847–851. 19020785

[23] Hao H, Wang G, Cui N, Li J, Xie L, et al (2007) Identification of a novel intestinal first pass metabolic pathway: NQO1 mediated quinone reduction and subsequent glucuronidation. Curr Drug Metab 8: 137–149. 17305492 10.2174/138920007779816011

[24] Wang Q, Hao H, Zhu X, Yu G, Lai L, et al (2010) Regioselective glucuronidation of tanshinone iia after quinone reduction: identification of human UDP-glucuronosyltransferases, species differences, and interaction potential. Drug Metab Dispos 38: 1132–1140. 20382756 10.1124/dmd.109.031864

[25] Liu F, Yu G, Wang G, Liu H, Wu X, et al (2012) An NQO1-initiated and p53-independent apoptotic pathway determines the anti-tumor effect of tanshinone IIA against non-small cell lung cancer. PLoS ONE 7: e42138. 22848731 10.1371/journal.pone.0042138 PMC3407158

[26] Hao H, Wang G, Cui N, Li J, Xie L, et al (2006) Pharmacokinetics, absorption and tissue distribution of tanshinone IIA solid dispersion. Planta Med 72: 1311–1317. 17024606 10.1055/s-2006-951698

[27] Bowalgaha K, Elliot DJ, Mackenzie PI, Knights KM, Miners JO (2007) The glucuronidation of Delta4-3-Keto C19- and C21-hydroxysteroids by human liver microsomal and recombinant UDP-glucuronosyltransferases (UGTs): 6alpha- and 21-hydroxyprogesterone are selective substrates for UGT2B7. Drug Metab Dispos 35: 363–370. 17151189 10.1124/dmd.106.013052

[28] Picard N, Ratanasavanh D, Premaud A, Le Meur Y, Marquet P (2005) Identification of the UDP-glucuronosyltransferase isoforms involved in mycophenolic acid phase II metabolism. Drug Metab Dispos 33: 139–146. 15470161 10.1124/dmd.104.001651

[29] Circu ML, Aw TY (2010) Reactive oxygen species, cellular redox systems, and apoptosis. Free Radic Biol Med 48: 749–762. 20045723 10.1016/j.freeradbiomed.2009.12.022 PMC2823977

[30] Siegel D, Ross D (2000) Immunodetection of NAD(P)H:quinone oxidoreductase 1 (NQO1) in human tissues. Free Radic Biol Med 29: 246–253. 11035253 10.1016/s0891-5849(00)00310-5

[31] Schlager JJ, Powis G (1990) Cytosolic NAD(P)H:(quinone-acceptor)oxidoreductase in human normal and tumor tissue: effects of cigarette smoking and alcohol. Int J Cancer 45: 403–409. 2307529 10.1002/ijc.2910450304

[32] Lewis AM, Ough M, Hinkhouse MM, Tsao MS, Oberley LW, et al (2005) Targeting NAD(P)H:quinone oxidoreductase (NQO1) in pancreatic cancer. Mol Carcinog 43: 215–224. 16003741 10.1002/mc.20107 PMC7262682

[33] Belinsky M, Jaiswal AK (1993) NAD(P)H:quinone oxidoreductase1 (DT-diaphorase) expression in normal and tumor tissues. Cancer Metastasis Rev 12: 103–117. 8375015 10.1007/BF00689804

[34] Bey EA, Bentle MS, Reinicke KE, Dong Y, Yang CR, et al (2007) An NQO1- and PARP-1-mediated cell death pathway induced in non-small-cell lung cancer cells by beta-lapachone. Proc Natl Acad Sci U S A 104: 11832–11837. 17609380 10.1073/pnas.0702176104 PMC1913860

