# Supplementary figures and images for: UDP-Glucuronosyltransferase 1A Compromises Intracellular Accumulation and Anti-Cancer Effect of Tanshinone IIA in Human Colon Cancer Cells
Source: PLoS One. 2013 Nov 14;8(11):e79172. doi: 10.1371/journal.pone.0079172 (PMC3828323; doi:10.1371/journal.pone.0079172)

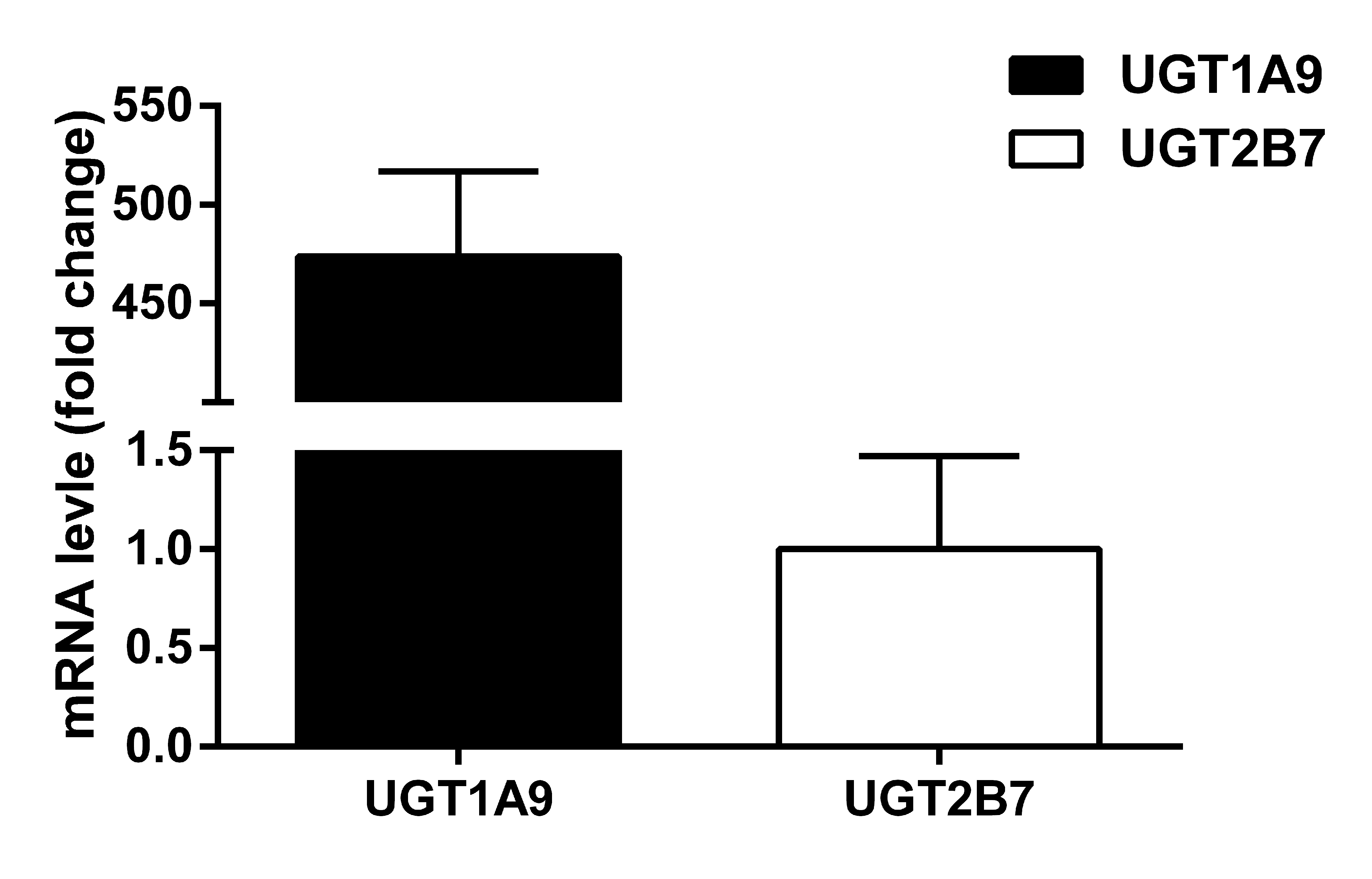

Supplement: Figure S1 — UGT1A9 and UGT2B7 mRNA levels in HT29 cells. mRNA levels were determined by RT-real time PCR. UGT2B7 mRNA level of HT29 cells were taken as 1. (TIF) [file pone.0079172.s001.tif]

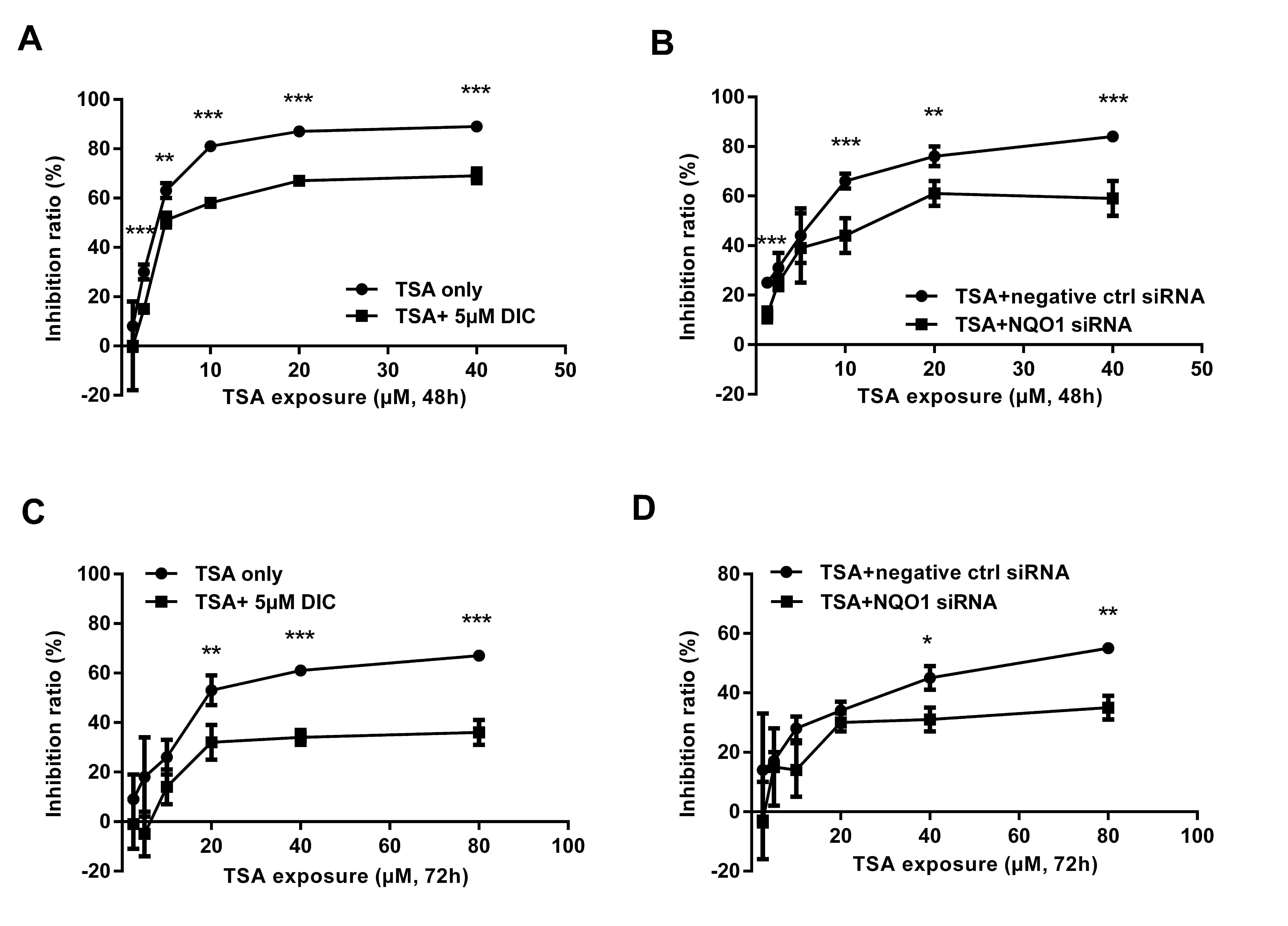

Supplement: Figure S2 — NQO1 affects TSA-induced cytotoxicity in HT29 and HCT116 cells. Cells were seeded by 5000/well in 96-well plate with NQO1 siRNA or non-specific siRNA (negative control) and cultured for 24 hours. Or cells were seeded by the same density, cultured for 24 hours, and pretreated with dicoumarol (DIC, Sigma, USA) for 2 hour. Then, Gradient concentrations of TSA (2.5–80 µM for HT29; 0.5–40 µM for HCT116) were added to cell culture medium and incubated for the indicated time and subsequently MTT assay was performed. (A) and (B) HCT116 cells; (C) and (D) HT29 cells. Results are presented as mean ± SD of at least four independent experiments (*P<0.05, **P<0.01, ***P<0.001). (TIF) [file pone.0079172.s002.tif]

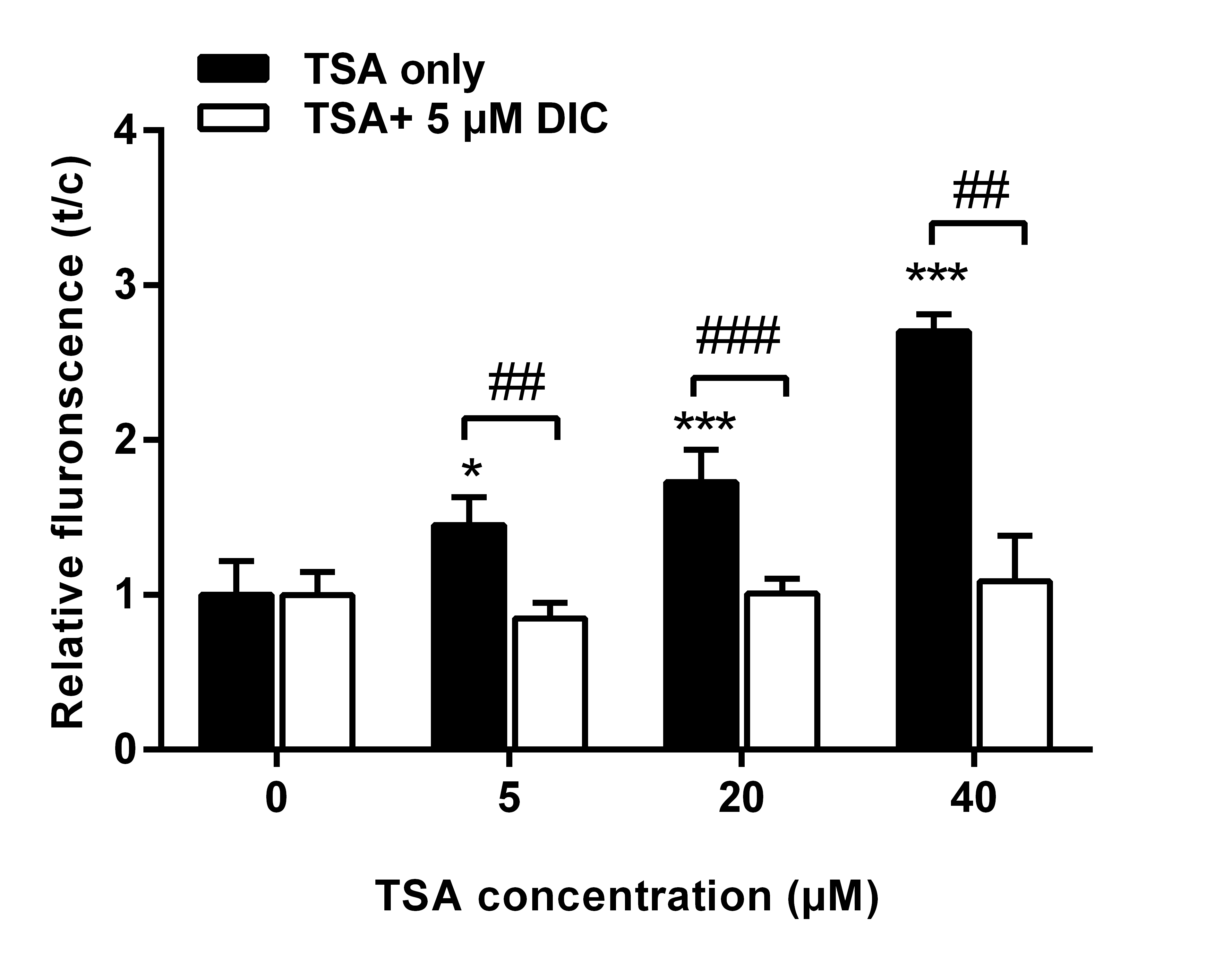

Supplement: Figure S3 — DIC inhibits TSA-induced ROS formation in HCT116 cells. Cells were pretreated with DIC (5 µM) for 2 hours. Then, cells were exposed to TSA (5, 20, 40 µM) for 1 h and subsequently treated by DCFH-DA. The fluorescence intensity was detected by a fluorimeter. Results are presented as mean ± SD of at least three independent experiments (*P<0.05, **P<0.01, ***P<0.001, TSA treatment vs control cells; #P<0.05, ##P<0.01, ###P<0.001, DIC pretreatment vs TSA only). (TIF) [file pone.0079172.s003.tif]

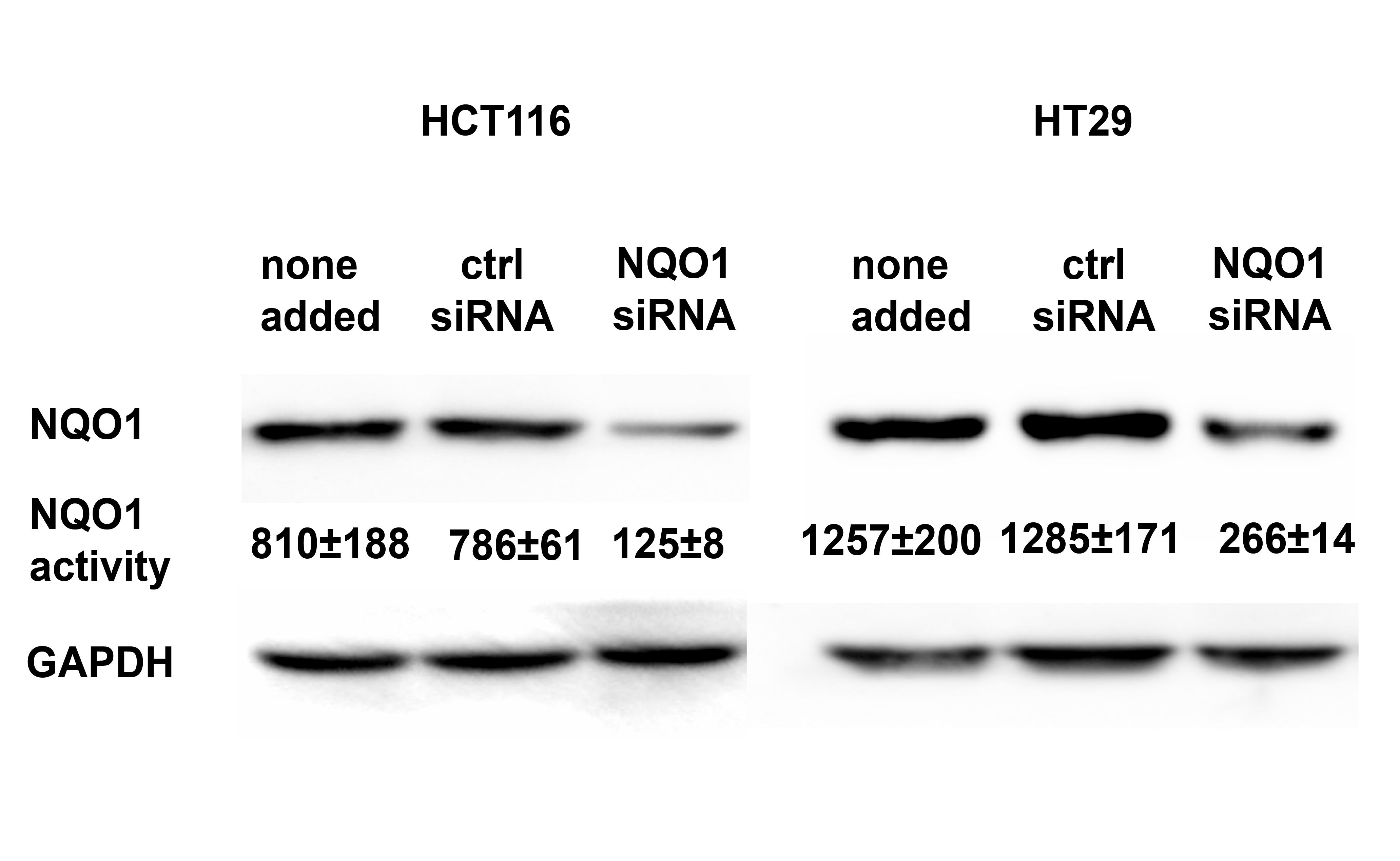

Supplement: Figure S4 — NQO1 protein levels and enzyme activities were determined in HT29 and HCT116 cells. NQO1 siRNA was used for NQO1-silence in both HT29 and HCT116 cells. Non-special siRNA was added as negative control. Specific NQO1 enzyme activity was determined as the rate of DIC-inhibitable 2, 6-Dichlorophenolindophenol (DCPIP, Sigma, USA) reduction in cell S9 fractions. The reaction was started by the addition of DCPIP, and the reduction of DCPIP was measured at room temperature at 600 nm by a microplate reader. The DIC-inhibitable part of DCPIP reduction was used to calculate NQO1 activity expressed as nmol DCPIP per minute per mg protein. Results are presented as mean ± SD of at least three independent experiments. (TIF) [file pone.0079172.s004.tif]
