# Supplementary material for: UDP-Glucuronosyltransferase 1A Compromises Intracellular Accumulation and Anti-Cancer Effect of Tanshinone IIA in Human Colon Cancer Cells
Source: PLoS One. 2013 Nov 14;8(11):e79172. doi: 10.1371/journal.pone.0079172 (PMC3828323; doi:10.1371/journal.pone.0079172)
Supplement: Table S1 — Sequences of the primers used in the study. (DOCX) [file pone.0079172.s005.docx]

**Table S1. Sequences of the primers used in the study**

| Primer | For | Rev |
| --- | --- | --- |
| UGT1A1 | 5’ CCTTGCCTCAGAATTCCTTC 3’ | 5’ ATTGATCCCAAAGAGAAAACCAC 3’ |
| UGT1A3 | 5’ TGTTGAACAATATGTCTTTGGTCTA 3’ | 5’ ACCACATCAAAGGAAGTAGCA 3’ |
| UGT1A6 | 5’ AGAGAATTTCTGCAGGGGTTTT 3’ | 5’ TTGGATTCTTTCAAAAGC 3’ |
| UGT1A9 | 5’ GAACATTTATTATGCCACCG 3’ | 5’ ATTGATCCCAAAGAGAAAACCAC 3’ |
| UGT1A10 | 5’ GAAAGCACAGGCACAAAGTATA 3’ | 5’ GGGAGGGAGAAATATTTAGCAAC 3’ |
| β-Actin | 5' CCTTCCTTCCTGGGTATG 3' | 5' TGTTGGCATAGAGGTCTT 3' |
